# Leading beyond the Job Description: Investigating the Association between Principals’ Leadership Styles and Teachers’ Organizational Citizenship Behaviors in North Wollo Secondary Schools

**DOI:** 10.12688/f1000research.183874.1

**Published:** 2026-07-20

**Authors:** Yihenew Belay, Mateb Tafere

**Affiliations:** 1 Educational Planning and Management, Bahir Dar University Faculty of Education and Behavioural Sciences, Bahir Dar, Amhara, Ethiopia

**Keywords:** Multifactor Leadership Theory; Secondary school; Extra-role Behaviors; Amhara Region; School Effectiveness

## Abstract

**Background:**

Unpredictable school leadership and a lack of participative decision-making hinder school effectiveness in Ethiopia, particularly in conflict-affected areas experiencing high teacher burnout and system disruptions. This study investigates how transformational, transactional, and laissez-faire leadership styles associate with teachers’ organizational citizenship behaviors in secondary schools of the North Wollo Zone, Amhara Region.

**Methods:**

An explanatory sequential mixed-methods design was employed. Quantitative data were collected from a proportionally selected sample of 334 teachers across 13 secondary schools using the Multifactor Leadership Questionnaire and a modified 24-item organizational citizenship behavior scale. This was followed by qualitative semi-structured interviews with seven purposively selected school principals to unpack contextual nuances. Quantitative data were analyzed using Pearson correlation and multiple linear regressions in SPSS20 software.

**Results:**

Transformational leadership was the most frequently practiced style (M = 2.88, SD = 0.55), followed closely by transactional leadership (M = 2.82, SD = 0.51), while laissez-faire scored lowest (M = 2.44, SD = 0.72). Teachers exhibited moderate overall organizational citizenship behaviors (M = 3.32, SD = 0.77). Correlation analysis revealed that transformational (r = .69, p < .001) and transactional (r = .61, p < .001) styles had significant positive associations with extra-role behaviors, while laissez-faire showed a strong negative correlation (r = −.55, p < .001). Multiple regression confirmed the model explained 63.8% of the variance, with transformational leadership acting as the strongest positive predictor (β = .39, p < .001) and laissez-faire as a severe negative predictor (β = −.38, p < .001).

**Conclusions:**

Active leadership styles are vital for fostering teachers’ organizational citizenship behaviors in challenging, conflict-affected environments. Educational training frameworks must integrate transformational competencies to maximize institutional effectiveness.

## 1. Introduction

School principals’ leadership has been shown to significantly influence institutional goals. This influence has implications for academic and behavioral outcomes ( Bledsoe, 2024; Daniels, 2023). While effective leadership is widely recognized as crucial, scholarly consensus indicates that no single leadership style is best for all situations. Effectiveness depends on specific organizational and environmental conditions ( Constantinou, 2025). Three interrelated topics form the basis of this study: the dominant leadership models in schools, their relationship with teacher Organizational Citizenship Behaviors (OCBs), and the contextual factors that affect these relationships. By identifying key literature gaps, particularly in under-researched settings like Ethiopia, this section provides a theoretical framework for this research.

Recent empirical studies cite positive teacher outcomes and school performance as the primary drivers of “positive transformation”. This transformation occurs when educators demonstrate a shared vision, encourage professional development, and actively participate in decision-making through leadership ( Fang & Yu, 2023). The presence of other configurations, such as transactional and distributed leadership, sows that schools need a diverse range of leadership styles ( Kearney, 2026). The Full Range Leadership Model (FRLM) and Multifactor Leadership Theory (MLT) encompass transformational, transactional, and laissez-faire leadership styles. These models provide a robust framework to explain their different impacts ( Constantinou, 2025). This central idea argues that the principal’s leadership style shapes school climate and teacher motivation. These in turn, drive the development of teachers’ OCBs ( Hermanto et al., 2024).

Teacher OCBs are discretionary actions beyond formal job duties, such as assisting colleagues, volunteering, and supporting school improvement. These behaviors remain critical to organizational and instructional effectiveness ( Fang & Yu, 2023). These actions contribute to a supportive environment, improve student outcomes, and buffer against counterproductive work behaviors. Consistent contemporary findings indicate that these OCBs are commonly preceded and nurtured by positive leadership behaviors ( Abdulrab, 2025; Hermanto et al., 2024). Furthermore, modern applications of Social Exchange Theory suggest that adaptive leadership is essential for fulfilling mutual obligations, and motivating employees to engage in constructive extra-role behaviors like OCBs ( Gedifew, 2025). Teacher OCBs are strongly encouraged by transformational leadership, while laissez-faire or passive-avoidant leadership actively inhibits their practice ( Constantinou, 2025; Fang & Yu, 2023). Conversely, research on transactional leadership remains varied: some studies show no significant correlation with extra-role efforts, while others suggest that strict compliance or rule-enforcing approaches can actually hinder discretionary initiatives ( Ingersoll et al., 2018; Kearney, 2026). The role of moderating variables is continually emphasized to explain these contradictions.

Leadership styles can have a significant impact on teacher behaviors due to various factors such as organizational culture, job satisfaction, reward systems, and wider socio-cultural values. Scholars emphasize that teachers’ beyond-contract contributions must be compatible with the distinct leadership style enacted within their specific school context ( Fang & Yu, 2023). Crucially, most existing research remains heavily concentrated in Western and certain Asian environments; there is a significant lack of research in African contexts, especially in Ethiopian educational settings ( Gedifew, 2025). In these environments, the unique socio-cultural, economic, and administrative dynamics may exert unforeseen impacts on the leadership-OCB dynamic.

The MLT, and FRLM, provide strong explanatory frameworks, but they are rarely deployed in these specific under-researched contexts to unpack the psychological processes linking principal leadership styles with teacher OCBs ( Gedifew, 2025). This research aims to test the Multifactor Leadership Theory framework within an updated educational paradigm ( Constantinou, 2025). This leadership model presents a matrix of three core leadership styles: transformational, transactional, and laissez-faire. This framework serves as a structured approach for analyzing complex leadership behaviors across varying levels of engagement and influence within the secondary schools of the Amhara Region’s North Wollo Zone in Ethiopia. Additionally, this framework addresses the contextual gap, aiming to resolve the central question: How do leadership styles relate to teacher OCBs in this particular study area? Ultimately, the objective is to go beyond a descriptive compilation and provide a contextually grounded, contemporary analysis of leadership in educational organizations.

### 1.1. Statements of the problem

In Ethiopia, the primary method of decision-making for the development of school leadership programs was dominated by political command. Unfortunately, these choices were not inclusive of educational stakeholders and frequently failed to incorporate the needs and concerns of the principals ( Gedifew, 2025; Tesfaye, 2018). Additionally, the evolution of school leadership was characterized by inconsistency, fluctuating rather than consistently advancing. Consequently, school leadership was not able to make the necessary contribution to the effectiveness of Ethiopian education ( Ministry of Education [MoE], 2023).

As the “new” Ethiopian education policy criticized not paying a high premium for relevance and quality ( Demeke, 2011), the MoE released an updated long-term education development plan to transform the education sector ( MoE, 2023). Regarding school leadership, this framework states that insufficient qualifications of administrative staff and partners in the secondary education sector are one of the major shortcomings. The official documentation also recommended that the Ministry of Education should reconsider the current administrative structure of school leadership in secondary education so that the problems with teacher management can be solved ( MoE, 2023). These recommendations indicate that the overall functioning of both school leadership and teachers was in a critical situation, which directly affected school effectiveness.

Recent international studies indicate that transformational leadership in principals and teachers’ OCBs has a significant positive correlation (r = .387, p < .005), suggesting consistent patterns across diverse global educational climates ( Fang & Yu, 2023; Sharma & Yadav, 2024). However, the generalizability of these international quantitative findings to the Ethiopian context is limited by several factors. Many of these foundational studies were conducted exclusively in urban areas within middle-income countries like Turkey or India, failing to consider the significant geographic, cultural, economic, and political differences unique to Ethiopia ( Assefa, 2024). Also, a sole reliance on quantitative methods in global literature prohibits the triangulation of results. Additionally, much of the existing sample data remains limited to elementary schools, where teacher conditions and academic standards differ significantly from those in secondary schools. Hence, it is important to exercise caution when applying external results to Ethiopia because there are significant differences in both context and method.

Conversely, some contemporary findings suggest that transformational and transactional leadership styles do not always directly affect teachers’ OCBs ( Arar & Abu-Nasra, 2020). This deviation from conventional literature may be due to a methodological limitation, particularly when measurement instruments fail to account for overlapping leadership styles and overall structural outcomes. These specific findings contradict a significant body of contemporary research linking OCB to transformational, transactional, or laissez-faire leadership paradigms ( Constantinou, 2025; Hermanto et al., 2024).

Locally, the performance of Ethiopian private higher educational institutions was best predicted by Markos and Bezabih (2022), who identified altruism as the most significant predictor due to sportsmanship, conscientiousness, and civic virtues. Nevertheless, Temesgen and Melaku (2022) conducted a qualitative study in Bahir Dar elementary schools and discovered varying scores for civic virtue, sportsmanship, conscientiousness, courtesy, and altruism. This contrast demonstrates how OCB practices and their perceived significance may vary significantly between educational levels (elementary vs. higher education) and contexts (urban vs. rural). Moreover, these distinctions highlight the overall problem that OCB in education is still not fully explored in developing countries, where cross-cultural differences in leadership and expectations are crucial for improved outcomes.

Another local study by Desta (2018), which solely examined the OCB dimensions of altruism, conscientiousness, and civic virtue, explicitly encouraged additional research to include the neglected aspects of sportsmanship and courtesy. This research seeks to bridge the divide and enhance universal applicability by examining the correlation between three principals’ leadership styles and all five OCB dimensions in secondary schools located in the North Wollo Zone of the Amhara Region. It concludes with relevant local studies demonstrating that the use of OCBs among teachers yields different outcomes within schools. This divergence can be attributed to various factors, such as insufficient teacher ownership ( Temesgen & Melaku, 2022) and the complex impact of modern principal leadership styles ( Fang & Yu, 2023). Also, insufficient leadership practices and inadequate governmental emphasis on secondary education contribute to this gap ( Assefa, 2024; Elias, 2020).

In order to address contextual and methodological gaps uncovered by the results, this research investigates the relationships and practices among principals’ leadership styles and teachers’ OCBs in Amhara State secondary schools within the North Wollo Zone during the 2024/2025 academic year. Therefore, the primary objective of this study is to investigate the leadership styles of principals and their corresponding Organizational Citizenship Behaviors (OCBs) of teachers in secondary schools within the North Wollo administrative Zone. The research will examine three key questions:

• What are the perceptions of leadership styles (transformational, transactional, and laissez-faire) among teachers regarding their principals in North Wollo Zone secondary schools?
• Is there a statistically significant relationship between principals’ leadership styles and teachers’ OCBs in North Wollo Zone secondary schools?
• Which leadership style most strongly predicts teachers’ OCB in secondary schools of the North Wollo Zone?

### 1.2. Objectives of the Study

The general objective of this study is to investigate the practices and association between principals’ leadership styles and teachers’ OCBs in North Wollo Zone secondary schools. Under this general objective, specific objectives include:

• To examine the practice of principals’ leadership styles (transformational, transactional, and laissez-faire) in North Wollo secondary schools, as perceived by teachers.
• To determine whether a statistically significant association exists between principals’ leadership styles and teachers’ OCBs in North Wollo Zone secondary schools.
• To identify the leadership style (transformational, transactional, or laissez-faire) that most strongly predicts teachers’ OCB in North Wollo secondary schools.

### 1.3. Conceptual framework

The conceptual framework of this study goes beyond the theoretical basis set in the introduction to examine how the dependent variable, teachers’ OCBs practices is related with independent variables identified as independent factors. According to the framework, which is based on established theories ( Bass, 1995; Organ, 1988), these styles are designed to predict voluntary and discretionary behaviors that are essential for school performance. It is anticipated that OCB will have a favorable impression of transformational leadership. OCB and job satisfaction in Africa are consistently positively linked to this style, which promotes intrinsic motivation and shared purpose ( Akullo & Kamanyire, 2023; Le, 2026; Liu et al., 2024; Mugizi et al., 2019). A mixed or weakly positive relationship is believed to exist between transactional leadership and teachers’ OCBs. While the contingent reward dimension can be beneficial, its management-by-exception dimension is often counterproductive and results in inconsistent empirical results ( Ludwikowska et al., 2024).

**Fig. 1.**
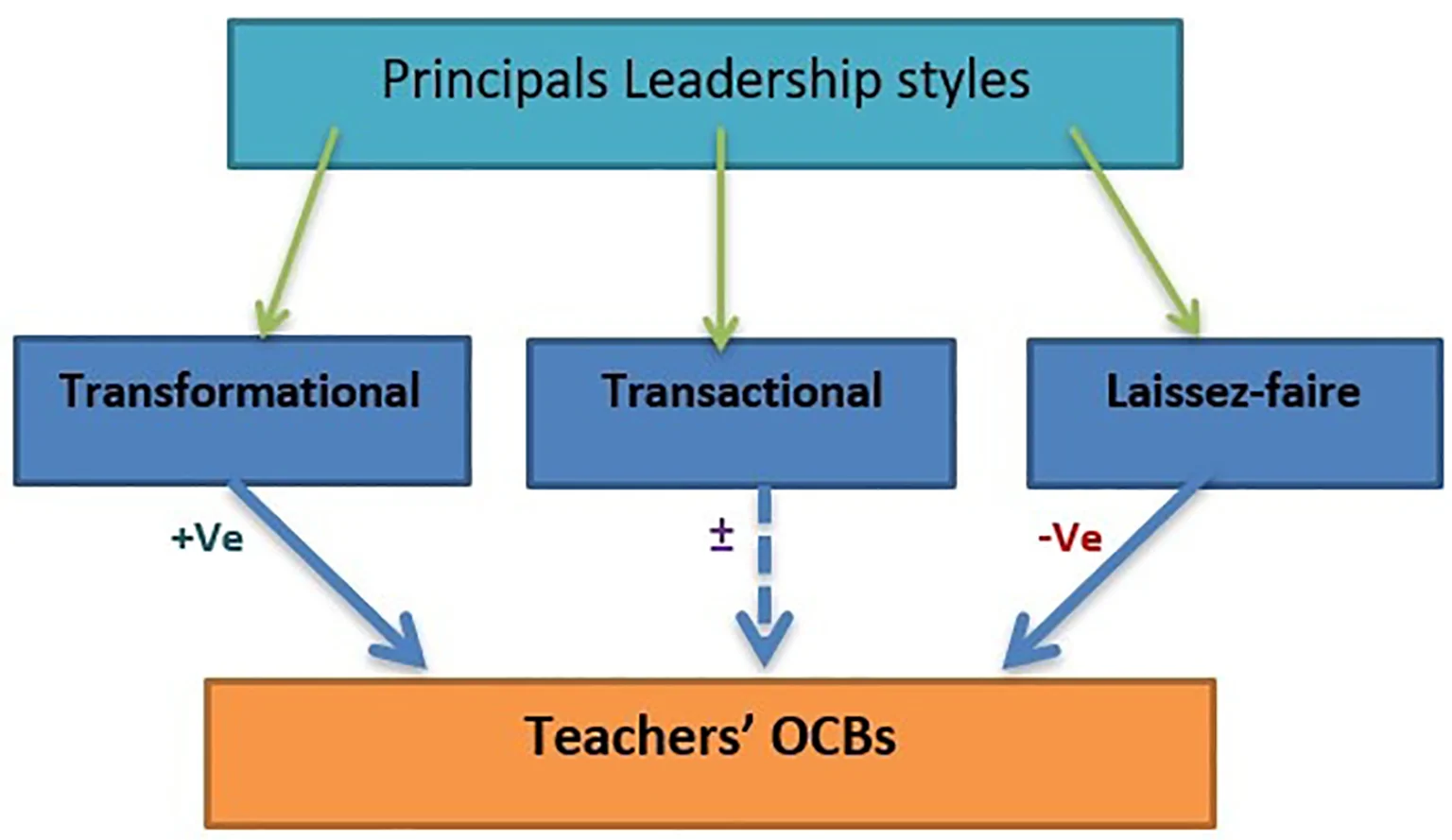
Conceptual Framework (Source: Literature review). This diagram maps the hypothesized predictive pathways between independent variables (Principals’ Transformational, Transactional, and Laissez-faire leadership styles) and dependent variable (Teachers’ Organizational Citizenship Behaviors) based on the Multifactor Leadership Theory and OCB Theory.

Teachers’ OCBs will reduce due to the laissez-faire leadership approach. This approach, as noted by McIntyre (2026) and Yohannes & Wasonga (2021), is associated with reduced staff motivation, a decreased sense of responsibility, and lower productivity due to the lack or absenteeism of school leaders (laissez-faire leadership style). Dimensions of OCBs are extensively used by contemporary researchers evaluating educational environments ( Adnan et al., 2026; Atış and Avcı, 2026; Öz, 2026). These include altruism, courtesy, sportsmanship, civic virtue, and conscientiousness. Additionally, the framework was based on theories of leadership and organizational behavior, which highlighted how various types or models of leadership can predict teachers’ OCBs that impact school performance ( Bass & Riggio, 2006; Organ, 2018). Based on these, the conceptual framework of this research suggests that transformational leadership has a positive impact on teachers’ OCB in North Wollo secondary schools, while transactional and laissez-faire leadership have weak and negative impact respectively (See Figure 1).

## 2. Research methods

### 2.1. Research design

The research was based on a pragmatic approach, as it is considered the philosophical cohort for the mixed methods approach. Pragmatism, as per the researchers, provides a framework for knowledge and promotes pragmatic approaches, which set it apart from post-positivism and constructivism ( Maxcy, 2003; Rallis & Rossman, 2003; Johnson & O'Neill, 2014). The researcher used an explanatory sequential mixed methods design (QUAN→qual). The design comprises of two steps: the quantitative data collection and analysis, followed by the qualitative phase based on numerical results of quantitative phase.

In quantitative part of the study, correlational design was employed to assess the strength, direction, and extent of the relationship ( Creswell, 2014) between the leadership styles of principals and the OCBs of teachers. Then, Qualitative case studies are chosen to provide a deeper understanding of why specific variables are significant predictors. Therefore, case selection was guided by examining a combination of typical cases and significant predictors ( Creswell and Creswell, 2018).

### 2.2. Population, sampling techniques and sample size

This research targeted a total population of 55 principals and 2522 teachers (1927 male and 595 female) from secondary schools located in the North Wollo administrative zone. The researcher conducted the quantitative phase using a multi-stage sampling method. The first phase involved categorizing schools into two clusters or strata (Urban and Rural) to ensure balanced representation. The second stage involved the selection of 13 secondary schools (5 urban and 7 rural) for each stratum, using a simple random sampling method. Using a proportional sampling technique, teachers were selected proportionally from each school that was selected in the second stage. The selection of sample teachers was done using Cochran’s (1977) formula, which is suitable for a large population, reduction of error, and accuracy in determining optimal sample size given. Therefore, 334 teachers were selected as a sample. Purposive sampling was used to select seven principals for an interview in the qualitative phase because experienced ones can provide discerning predictions about their leadership style and staff’s OCB. (See Table 1).

**Table 1.** Demographic characteristics of respondent teachers.

Characteristic	Category	Frequency (n)	Valid Percent (%)
Sex	Male	257	84.8
	Female	46	15.2
Residence	Urban	169	55.8
	Rural	134	44.2
Qualification	Diploma	5	1.7
	BA	123	40.6
	BSc	85	28.1
	MA	56	18.5
	MSc	34	11.2

*Note*: Table 1 displays the frequencies and valid percentages for the primary demographic variables of the teacher sample (N = 303).

### 2.3. Data collection instruments

By using the Multifactor Leadership Questionnaire (MLQ-5X rater form) developed by Avolio & Bass (2004), teachers rated the leadership styles of the principals. The MLQ-5X (rater-form) was chosen because it provides an edge by emphasizing the different leadership types, leading to a more precise assessment and more leadership development strategies ( Gougas & Malinova, 2021), and is extensively proven in educational settings ( Johnson & Onwuegbuzie, 2004; Tigist, Zenebe, & Belete, 2023). This instrument examines three types of leadership styles (transformational, transactional and laissez-faire) at the same time. MLQ comprises 45 questions on a five-point scale of 0 to 4, (0 = not at all to 4 = frequently, if not always).

This study utilized Podsakoff, Ahearne & MacKenzie (1997) 24-item OCB scale that was later modified by Herren (2014). This instrument examines the five fundamental aspects of OCBs (altruism, conscientiousness, sportsmanship, courtesy, and civic virtue). Teachers used a five-point Likert scale to determine their level of agreement with each statement, starting from “Strongly Disagree” (1) and ending at “Slightly Agree” (5). The semi-structured interview that was self-prepared was used to gather information from principals on their leadership styles and teachers’ OCBs in their schools.

### 2.4. Validity and reliability

In the pilot study phase, all instruments used to gather data were thoroughly tested for their validity. The internal consistency of the research instrument was confirmed in a pilot test with 90 teachers from Millennium Secondary School in Woldia town, where high Cronbach’s Alpha coefficients for transformational leadership style (α =0.97), transactional Leadership style (α =0.88), laissez-faire leadership (α =0.87) and teachers’ organizational citizenship behavior (α =0.89) were confirmed. These values are validated in previous studies. For example, Research on OCB has revealed an alpha coefficient of α = 0.75 ( Niazi 2005) and α =0.94 ( Karabatak, Alanoglu & Sebgur 2018).

In the main investigation, the reliability was verified and produced similarly robust outcomes for transformational, transactional, laissez-faire leadership styles and teachers’ OCBs (α =0.96, 0.87, 0.88, and 0.909, respectively), with internal consistency that ranged from satisfactory to high across all variables. This high-reliability finding indicates that the items within each construct accurately accounted for their intended theoretical domains (Pallant, 2020), providing a strong empirical basis for the study.

### 2.5. Data Analysis techniques and procedures

The surveyed quantitative data were analyzed by using descriptive and inferential analysis. Descriptive statistics were employed to analyze the quantitative data for frequencies, averages and patterns describing the demographic data of the participants. Mean values and standard deviations were also used to examine opinion, and the extent of implementations of PLS and TOCB in sample schools. Inferential statistics were employed to compare mean differences among respondent groups regarding the variables’ practices, analyze their relationships, and determine the predictive power of the independent variables on the dependent variable.

Pearson Product-moment correlation analysis was employed using SPSS version 20 and tested using a level of significance of 0.05. Hence, it allows testing of the strength and direction of the relationships between scores obtained and showing the predictability of variables (PLS & OCB) ( Creswell, 2014). Multiple linear regressions was employed to see which independent variable (Transformational leadership, Transactional leadership, and Laissez-fair leadership) has the highest Beta coefficient, signifying it has the most influence on to the dependent variable (TOCB).

To determine the internal consistency, linearity, and multicollinearity of the instrument, it was first tested for normality (Isacco & Mores, 2015) before analysis of main data. In order to assess missing values, outliers, and errors, we examined the skewness and kurtosis coefficient (Leithwood and Sun, 2012). Both descriptive and inferential statistical analyses were conducted using SPSS version 20 software (See appendices).

## 3. Results

In the sample secondary schools, 334 teachers received survey questionnaires. There were 391 (95.51%) questionnaires that had to be returned, of which 303 (94.98%) were correctly completed, and the remaining 16 (5.02%) were partially filled and not completed as intended and rejected. 7 secondary school principals were interviewed in a semi-structured manner for each study context (see Table 1).

### 3.1. Demographic characteristics of participants

The 303 teacher respondents’ key demographic variables are presented in Table 1. 84.8 per cent of the sample was comprised of males, while urban school population made up 55.8%. The majority of teachers were graduated with either a BA (40.6%) or BSc (28.1%). Among those with teaching experience, the majority had 11–15 years (31.0%).

### 3.2. Status of principals’ leadership styles

Table 2 shows that the prevalence of transformational leadership (M = 2.88, SD = 0.55) was higher than either transactional leaders (M = 2.82, SD = 0.51) or laissez-faire (M = 2.44, SD = 0.72) leadership styles.

**Table 2.** Descriptive statistics of leadership styles.

Dimension	N	Mean	SD
Transformational Leadership	**303**	**2.88**	**0.55**
Idealized Attributes	303	2.89	0.54
Idealized Behaviors	303	2.92	0.52
Inspirational Motivation	303	2.84	0.55
Intellectual Stimulation	303	2.91	0.56
Individual Consideration	303	2.79	0.56
Transactional Leadership	**303**	**2.82**	**0.51**
Contingent Reward	303	2.86	0.50
Management-by-Exception/Active	303	2.79	0.53
Laissez-Faire Leadership	**303**	**2.44**	**0.72**

*Note*: SD = Standard Deviation. Table 2 displays Mean scores and standard deviations for the three core leadership styles and their respective sub-dimensions as perceived by secondary school teachers (N = 303).

For transformational leadership, all dimensions show mean scores consistently around the 2.8 to 2.9 range. Specifically, Idealized Behaviors (M = 2.92, SD = 0.52) and intellectual stimulation (M = 2.91, SD = 0.56) exhibit higher mean scores, suggesting that leaders are perceived as embodying desired values and encouraging innovative thinking more often. Idealized Attributes (M = 2.89, SD = 0.54) and Inspirational Motivation (M = 2.84, SD = 0.55) also indicate a moderate practice of leaders serving as role models and inspiring followers. Individual Consideration (M = 2.79, SD = 0.56) shows the lowest mean among the transformational dimensions, implying that personalized attention to followers’ needs is somewhat less prevalent. Relatively small standard deviations across these dimensions suggest a consistent level of practice for each.

Within transactional leadership, contingent reward (M = 2.86, SD = 0.50) is practiced more frequently than management by exception/Active (M = 2.79, SD = 0.53). This indicates that leaders are more likely to provide rewards for achieving goals and clarify expectations, rather than actively monitoring for deviations from standards. The third leadership style is further underscored by the Laissez-faire leadership style, which exhibits the lowest overall mean (M = 2.44, SD = 0.72) among all leadership styles and their dimensions. This indicates a general tendency for leaders to avoid responsibility and decision-making less often, which is a positive sign for organizational engagement.

### 3.3. Status of teachers’ OCBs

As described in Table 3, secondary school teachers engaging in OCBs at a moderate level (M = 3.32). The mean scores across the dimensions highlights some interesting differences. Sportsmanship (M = 3.43, SD = 0.70) and Courtesy (M = 3.36, SD = 0.71) received the highest mean scores, suggesting that teachers are more likely to demonstrate a positive attitude in the face of minor annoyances and to take preventative steps to avoid problems with colleagues. These findings imply a relatively harmonious and resilient work environment where teachers are willing to endure less-than-ideal circumstances without complaining and are proactive in their interpersonal interactions. This is a positive indicator for overall workplace climate and collegiality.

**Table 3.** Descriptive statistics of teachers OCBs.

Variable	Dimensions	N	Mean	Std. Deviation	Standard Error Mean	95% Confidence Interval of the Difference	
						Lower	Upper
TOCB	Altruism	303	3.3408	0.7811	0.04487	3.2525	3.4291
	Courtesy	303	3.3584	0.70757	0.04065	3.2784	3.4384
	Conscientiousness	303	3.1710	0.82571	0.04744	3.0776	3.2643
	Sportsmanship	303	3.4277	0.69931	0.04017	3.3487	3.5068
	Civic Virtue	303	3.3091	0.84580	0.04859	3.2135	3.4047
	**Overall TOCB**	303	**3.3214**	**0.7742**	**0.04448**	**3.2342**	**3.4086**

*Note*: Table 3 displays Means and standard deviations across the five fundamental dimensions of organizational citizenship behavior among the sample faculty (N = 303).

Conversely, the Conscientiousness dimension produced the smallest average score (M = 3.17, SD = 0.83). This implies that teachers may not always be responsible for meeting standards, being punctual, and meticulous in their work. Although still in the moderate range, this lower score could be a potential area for professional development and training. At a moderate level, teachers did demonstrate some kind behavior toward coworkers and participate in the school community, although not as often as sportsmanship or civic virtue. Additionally, Altruism (M = 3.34, SD = 0.78) and Civic Virtue (M = 3.31, SD = 0.85) were observed at an average level.

### 3.4. Association between leadership styles and teachers’ OCBs

Table 4 revealed that a significant positive correlation existed between teachers’ OCBs and transformational leadership style (r = .69, p < 001). A comparison of the two outcomes was also noteworthy. Teachers who view their principals as more influential are likely to have higher levels of OCBs, according to the findings. A strong positive association for transactional leadership was also observed (r = .61, p < 001). In comparison, there was a strong negative correlation (r = −.55, p < 001) for laissez-faire leadership. Conversely, inter-leadership correlations indicate that transformational and transactional leadership styles are frequently coexisting (r = 0.682, p < .001), and both exhibit negative associations with laissez-faire leadership, although not strongly. The transformational (r = −0.303) is highly challenging in contrast to the transactional (r = − 0.197).

**Table 4.** Correlation matrix for leadership styles and teachers OCBs.

Variable	1	2	3	4
Teacher OCB	—			
Transformational	.69 **	—		
Transactional	.61 **	.68 **	—	
Laissez-Faire	−.55 **	−.30 **	−.20 **	—

*Note*: Table 4 displays Pearson Product-Moment correlation coefficients indicating cross-variable linear associations (N = 303).

** *p < .001*

### 3.5. Predictive power of principals’ leadership styles on teachers’ OCBs

The use of multiple regression analysis was necessary to determine the distinct role that each leadership style played in accounting for variance in teacher OCB. This model was important, accounting for about 64 percent of the variance in OCB (Adjusted R ^2^ = .634).

As presented in Table 5, the predictor variables, transformational, transactional and laissez-faire leadership, exhibit a positive correlation between the dependent variable and teachers’ OCB, with R values of.799. The high R value indicates that these leadership styles are effective in forecasting differences in Teachers’ OCB. A linear combination of the three leadership styles included in the model accounts for 63.8% (the overall variation) of Teachers’ OCBs. Adjusted R ^2^ (.634) is a conservative approximation of the population R ^2^, which takes into account the number of predictors in that model and also the size of this sample. The Standard Error of the Estimate is.37463. The included leadership styles can accurately predict teachers’ OCB, with a precision of.37463 in this case. The Durbin-Watson statistic reaches 1.933. Autocorrelation is identified in the residuals (errors) of a regression analysis by means of this statistic.

**Table 5.** Multiple regression analysis.

Model Summary b					
Model	R	R Square	Adjusted R squares	Std. error of the estimates	Durbin-Watson
	.799 ^a^	.638	.634	.37463	1.933

*Note*: Table 5 displays Regression framework summary highlighting the cumulative explanatory power and fit parameters of the leadership styles model.

^a^ Predictors: (Constant), Laissez-Fair, Transactional, Transformational

^b^ Dependent Variable: Teachers OCB

The absence of significant autocorrelation is indicated by a value near 2. However, 1.933 is close to 2, which means that the assumption of independent errors is fulfilled, and there’s no need to worry about whether the residuals have positive or negative correlation. The regression findings become more dependable. In summary, the model demonstrates that the regression model has a high explanatory power and is strong predictor of teachers’ OCB with sufficiently satisfying statistical assumptions.

In addition, Table 6 exhibited that transformational leadership was a statistically significant positive predictor of teachers’ OCB (B = 0.464, t = 7.853, p < .001). This implies that, for every one-unit increase in transformational leadership, teachers’ OCB is predicted to increase by 0.464 units, holding all other predictors constant. The standardized beta coefficient (β = 0.385) indicates that transformational leadership has a substantial unique contribution to explaining the variance in teachers’ OCB.

**Table 6.** Regression analysis summary.

Predictor	B	SE	β	t	p
(Constant)	1.88	0.18		10.31	<.001
Transformational	0.46	0.06	.39	7.85	<.001
Transactional	0.35	0.06	.27	5.72	<.001
Laissez-Faire	−0.35	0.03	−.38	−10.38	<.001

*Note*: Dependent Variable = Teacher OCB. VIF values ranged from 1.10 to 1.98, indicating no critical multicollinearity.

Table 6 displays Standardized (β) and unstandardized (B) regression weights detailing the unique predictive capabilities of individual leadership styles on teacher OCBs.

Transactional leadership also emerged as a statistically significant positive predictor of teachers’ OCB (B = 0.349, t = 5.716, p < .001). A one-unit increase in transactional leadership is associated with a 0.349-unit increase in teachers’ OCB, assuming other variables remain constant. The standardized beta coefficient (β = 0.272) suggests a notable unique contribution of transactional leadership to teachers’ OCB, though slightly less than that of transformational leadership.

Conversely, laissez-fair leadership was a statistically significant negative predictor of teachers’ OCB (B = −0.352, t = −10.378, p < .001). This indicates that for every one-unit increase in laissez-fair leadership, teachers’ OCB is predicted to decrease by 0.352 units, holding other predictors constant. The standardized beta coefficient (β = −0.379) suggests that laissez-fair leadership has a strong negative unique contribution to teachers’ OCB.

The Collinearity statistics (Tolerance and VIF) indicate that multicollinearity is not a significant concern in this model. All tolerance values are above 0.1, and all VIF values are well below 10 (Transformational VIF = 1.980; Transactional VIF = 1.870; Laissez-Fair VIF = 1.102). These values suggest that the independent variables are not overly correlated with each other, allowing for reliable interpretation of their individual effects on the dependent variable.

Semi-structured interviews results suggested the main causes of reduced teachers OCB dimensions in the conflict affected areas, North Wollo administrative Zone. Teaching is often discouraged from helping others because of stress, exhaustion, and survival concerns. Conscientiousness has led to a decrease in punctuality, preparation time, and voluntary effort, resulting in system disruptions and teachers burnout. Furthermore, conflict affected courtesy heightened with staff members who experienced stress, distrust, and anxiety. The existing conflict also affects teachers’ sportsmanship by making more frustration, complaints, pessimism, and a lack of tolerance for hardship. Due to insecurity and hopelessness, individual teacher exhibit reduced participation in meetings, committees, and school improvement activities (civic virtue).

## 3. Discussions, conclusions and implications

RQ1: What are the perceptions of leadership styles among teachers regarding their principals in North Wollo Zone secondary schools?

The research revealed that transformational leadership style was the most frequently practiced leadership style among principals (M = 2.88, SD = 0.55), followed by transactional leadership style (M = 2.82, SD = 0.51), while laissez-faire leadership style exhibited the lowest mean score (M = 2.44, SD = 0.72). This implies that principals in the sampled secondary schools were more inclined toward active leadership approaches that involve motivating teachers, providing guidance, and maintaining organizational structures rather than avoiding responsibilities or remaining passive in administrative duties. The relatively higher prevalence of transformational leadership supports the arguments of Constantinou (2025), Fang and Yu (2023), and Hermanto et al. (2024), who emphasized that modern school leadership increasingly depends on inspiring shared vision, professional support, and teacher participation in decision-making processes. This finding also aligns with Bass and Riggio’s (2006) FRLM, which considers transformational leadership as the most effective type of leadership in educational settings because it enhances subordinates’ intrinsic motivation and organizational commitment which elevate teachers’ OCBs.

One possible explanation for the dominance of transformational leadership in North Wollo secondary schools may be linked to the increasing national emphasis on educational reform and school improvement in Ethiopia. The Ministry of Education ( MoE, 2023) strongly recommended strengthening leadership quality and improving teacher management practices. As a result, principals may attempt to adopt more participatory and motivational leadership behaviors to address institutional challenges, teacher dissatisfaction, and declining school effectiveness.

The finding also showed that transactional leadership was practiced at a moderately high level. This suggests that principals frequently use contingent rewards, supervision, and corrective mechanisms alongside transformational practices. Such coexistence between transformational and transactional leadership styles is consistent with the Multifactor Leadership Theory (MLT), which argues that effective leaders often combine multiple leadership approaches depending on organizational demands ( Bass, 1995; Constantinou, 2025). Similarly, Kearney (2026) noted that schools require a combination of leadership styles because educational environments are complex and demand both inspiration and accountability.

The existence of transformational and transactional leadership styles observed in this study may also reveal the realities of Ethiopian secondary schools, where principals are expected not only to motivate teachers but also to ensure obedience with administrative rules, regulations, performance standards, and organizational directives. This combination therefore seems contextually appropriate.

On the contrary, laissez-faire leadership style scored the lowest mean, indicating that passive and avoidant leadership behaviors were less common. This result supports previous studies by McIntyre (2026) and Yohannes and Wasonga (2021), who found that laissez-faire leadership is generally undesirable in educational settings because it reduces staff motivation, weakens accountability, and creates organizational confusion. The lower prevalence of laissez-faire leadership may indicate that principals recognize the urgent need for active leadership in schools experiencing educational and social challenges, particularly within conflict-affected areas like North Wollo.

Regarding teachers’ OCBs, the average score (M = 3.32, SD = 0.77) shows that teachers generally practice OCBs at a moderate level. Among the different areas, sportsmanship and courtesy had the highest average scores, but conscientiousness had the lowest score. The higher level of sportsmanship shows that teachers are usually ready to put up with awkward situations at work and stay positive even when things are tough. Teachers seemed to show a good measure of courtesy, which suggests they try to keep good relationships with their colleague and avoid disagreements. These results might be linked to the collectivist culture often seen in Ethiopian communities, where people place a lot of importance on respecting others, working together, and keeping things peaceful and in balance.

On the other hand, conscientiousness demonstrated comparatively lower levels. This may reflect the difficult working conditions challenging teachers in North Wollo Administrative zone, particularly in conflict-affected environments. The qualitative interview findings strongly support this interpretation, revealing that stress, burnout, insecurity, and system disruptions reduced punctuality, preparation, and voluntary work effort among teachers. These findings are consistent with Temesgen and Melaku (2022), who emphasized that insufficient teacher ownership and difficult working conditions negatively affect OCB practices.

The moderate level of altruism and civic virtue shows that teachers do take part in helping others and school activities, but they don’t do it at very high levels all the time. This somewhat agrees with Markos and Bezabih (2022), who discovered that altruism is a major factor in how well institutions perform in Ethiopian higher education. However, the current results are a bit different because this study focused at secondary schools in a rural area affected by conflict, instead of higher education institutions in more stable urban areas. The differences between this research finding and some earlier studies may therefore be attributed to contextual variations such as educational level, school environment, socio-economic conditions, and the ongoing conflict situation in North Wollo. This confirms the argument presented by Gedifew (2025) that leadership and OCB relationships are highly context-dependent and cannot always be generalized across countries or institutional settings.

The findings contribute significantly to understanding the phenomenon under study by demonstrating that leadership practices and teachers’ OCBs are shaped not only by organizational structures but also by contextual realities such as conflict, stress, and institutional instability. The results extend the applicability of the FRLM and Social Exchange Theory within Ethiopian secondary school settings, an area previously underrepresented in leadership research.

Practically, the findings imply that educational authorities should strengthen leadership development programs that encourage transformational leadership behaviors while simultaneously improving teacher working conditions. Greater institutional support, professional development, and psychological support mechanisms may help enhance teachers’ conscientiousness and other forms of OCB.

RQ2 : Is there a statistically significant relationship between principals’ leadership styles and teachers’ OCBs?

The correlation analysis revealed statistically significant relationships between principals’ leadership styles and teachers’ OCBs. Transformational leadership style demonstrated a strong positive correlation with teacher OCB (r = .69, p < .001), transactional leadership style also showed a strong positive relationship (r = .61, p < .001), while laissez-faire leadership style had a strong negative relationship with teacher OCB (r = −.55, p < .001). This strong positive association strongly supports the assumptions of Social Exchange Theory and FRLM. According to SET, employees reciprocate supportive and motivational leadership behaviors through positive discretionary actions such as helping colleagues, volunteering, and participating actively in organizational activities ( Gedifew, 2025). Therefore, when principals demonstrate inspirational motivation, individualized consideration, and intellectual stimulation, teachers are more likely to respond with higher levels of OCB.

This finding is highly consistent with previous studies conducted by Fang and Yu (2023), Sharma and Yadav (2024), Hermanto et al. (2024), and Constantinou (2025), all of which reported positive and significant relationships between transformational leadership and teachers’ OCBs. The current result therefore strengthens the growing body of evidence suggesting that transformational leadership style consistently promotes positive teacher behaviors across diverse educational contexts.

The findings also demonstrated that transactional leadership had a significant positive relationship with teacher OCBs. This result partially supports Bass and Riggio’s (2006) argument that contingent reward mechanisms may encourage positive employee behaviors when implemented appropriately. Teachers may have positive response to clear expectations, recognition, and fair supervision because such practices provide organizational structure and predictability. However, these findings differ somewhat from studies such as Ingersoll et al. (2018) and Kearney (2026), which reported inconsistent or weak relationships between transactional leadership style and OCBs. One possible explanation for this difference may lie in contextual realities within Ethiopian secondary schools. In environments characterized by resource limitations, institutional instability, and administrative challenges, teachers may appreciate leadership behaviors that provide clear direction, accountability, and organizational order. Consequently, transactional leadership may become more positively valued than in contexts where teacher autonomy is already well established.

The strong negative relationship between laissez-faire leadership and teachers’ OCBs is also consistent with previous empirical findings ( Constantinou, 2025; Fang & Yu, 2023; Yohannes & Wasonga, 2021). Passive leadership weakens organizational trust, reduces motivation, and creates uncertainty within schools. Teachers who perceive their principals as absent or uninvolved are less likely to engage voluntarily in extra-role behaviors because they may feel unsupported, neglected, or disconnected from organizational goals. The interview findings provide further explanation for this relationship. Participants reported that conflict, stress, exhaustion, insecurity, and hopelessness reduced teachers’ willingness to participate in school improvement activities and weakened dimensions such as civic virtue, courtesy, and conscientiousness. In such challenging environments, passive leadership may increase organizational instability and further discourage teachers from engaging in OCBs.

The strong relationships between leadership styles and OCBs identified in this study contribute theoretically by reinforcing the relevance of the Multifactor Leadership Theory and Social Exchange Theory in explaining teacher behavior within under-researched African educational contexts. The study demonstrates that leadership styles significantly shape teachers’ willingness to perform beyond formal job requirements even in challenging and conflict-affected settings.

The findings also contribute practically by highlighting the importance of active and supportive school leadership. Educational policymakers and training institutions should prioritize leadership preparation programs that emphasize transformational leadership competencies such as motivation, communication, emotional support, and participatory decision-making. Reducing passive leadership behaviors may also improve teacher morale, organizational climate, and overall school effectiveness.

RQ3 : Which leadership style most strongly predicts teachers’ OCBs in North Wollo secondary schools?

The regression analysis revealed that transformational leadership was the strongest positive predictor of teachers’ OCBs (β = .39, p < .001), followed by transactional leadership (β = .27, p < .001). In contrast, laissez-faire leadership significantly predicted lower teacher OCBs (β = −.38, p < .001). The overall regression model explained approximately 63.8% of the variance in teachers’ OCBs (Adjusted R ^2^ = .634), indicating substantial explanatory power. This finding is highly consistent with the theoretical assumptions of the Full Range Leadership Model and with earlier empirical findings ( Fang & Yu, 2023; Hermanto et al., 2024; Sharma & Yadav, 2024). Transformational leaders create emotional attachment, collective commitment, and professional inspiration among teachers, which naturally encourages OCBs.

The predictive power of transformational leadership may be explained by its ability to satisfy teachers’ psychological and professional needs simultaneously. Teachers who feel respected, supported, and empowered are more likely to exceed formal expectations and contribute voluntarily to school success. This interpretation aligns with Social Exchange Theory, which suggests that supportive organizational relationships motivate reciprocal positive behavior.

Transactional leadership style also significantly predicted teachers’ OCBs, though less strongly than transformational leadership. This suggests that reward systems, monitoring, and administrative control still play important roles in shaping teacher behavior. In contexts where educational resources are constrained and institutional accountability is essential, teachers may respond positively to leadership practices that establish order and recognize performance. However, the comparatively weaker predictive power of transactional leadership supports earlier arguments that transactional approaches alone may not fully sustain intrinsic motivation or long-term organizational commitment ( Ludwikowska et al., 2024). Teachers may comply with institutional expectations under transactional leadership, but transformational leadership appears more effective at encouraging voluntary, emotionally driven organizational behaviors.

The strong negative predictive outcome of laissez-faire leadership style further confirms that passive leadership damages organizational effectiveness. When principals fail to provide direction, support, or supervision, teachers may experience frustration, uncertainty, and reduced institutional attachment. This finding strongly aligns with McIntyre (2026) and Yohannes and Wasonga (2021), who emphasized that laissez-faire leadership weakens motivation and efficiency within schools.

The relatively high explanatory power of the regression model (Adjusted R ^2^ = .634) suggests that leadership styles are major determinants of teachers’ OCBs in North Wollo secondary schools. Nevertheless, approximately 36% of the variance remained unexplained, implying that additional variables such as organizational culture, workload, teacher satisfaction, conflict exposure, salary conditions, and professional development opportunities may also influence OCBs.

The findings contribute to theory by validating the predictive relevance of leadership theories within Ethiopian secondary school contexts. They also extend contemporary leadership literature by demonstrating that transformational leadership remains effective even within conflict-affected and resource-constrained educational environments.

From a practical perspective, the findings suggest that educational leadership training in Ethiopia should prioritize transformational leadership competencies. School principals should be trained to motivate teachers, communicate shared visions, provide emotional and professional support, and encourage participatory school cultures. Simultaneously, policymakers should discourage passive leadership practices and strengthen administrative accountability systems.

Generally, the study contributes significantly to understanding how principals’ leadership styles shape teachers’ willingness to engage in discretionary organizational behaviors within Ethiopian secondary schools. By addressing contextual gaps in African educational leadership research in general North Wollo Zone secondary schools leadership in particular, the findings provide valuable insights for researchers, policymakers, and practitioners seeking to improve school effectiveness through leadership development and teacher engagement strategies.

## 4. Limitations

There were some limitations in this study. The absence of causal relationships between leadership styles and OCB is due to its cross-sectional design. Trust on self-reported data introduces the possibility of biases towards common methods and social ‘desirability’ effects. Additionally, because the sample was taken from a single administrative zone, it is possible that results could be generalized across other regions with different cultural or operational environments.

## 5. Recommendations

The outcomes suggest that to improve principals’ leadership style and teacher OCBs and overall school effectiveness in North Wollo Zone and similar settings, the following steps should be taken:

• The integration of transformational and active transactional leadership proficiencies into the lifecycle of school management, particularly within recruitment, training, and performance evaluation frameworks, is a crucial aspect that educational governing bodies should consider to maximize institutional effectiveness.
• In order to achieve systemic integration, state-funded professional development must prioritize practical skill acquisition through instructional coaching, persuasive communication, and equitable management in stressful environments such as Amhara regional State in general and North Wollo Administrative Zone in particular.
• Furthermore, anonymous, data-driven school climate surveys are required to provide an unbiased view of teacher attitudes, which will enable districts to link administrative policy with the actual organizational reality of the classroom.
• The key to achieving successful educational organizations is, for school leaders, to articulate a strong organizational vision that care for innovative pedagogy while also maintaining commitment to personal guidance and support. To support this innovative strategy, it is crucial to establish transparent standards for teaching and administration obligations as well as a uniform system of merit-based acknowledgment of educational effectiveness.
• Effective leadership in schools demands a balance between active presence and decisive accountability. By offering structured guidance and being visible, especially to teachers with less experience, principals can prevent the potential risks of perceived detachment and encourage meaningful collective growth through shared responsibilities.
• To enhance the understanding of teacher engagement in Ethiopian education, future studies should adopt longitudinal and experimental methods that enable a stronger causal inference, particularly by examining OCB surrounding structured leadership interventions.
• It will be important to broaden the scope of this research to include different contexts within Ethiopia and educational levels in order to make these conclusions more general and identify unique moderating contextual variables.
• Also, by utilizing diverse influences (such as teacher self-efficacy, resource availability and community dynamics), scholars can develop a more holistic and humanistic approach to the professional commitment of teachers that considers the complex realities of the teaching profession in developing countries.

## Ethical approval statement

This study was approved by the Institutional Review Board (IRB) of College of Education and Behavioral Science, Bahir Dar University, Ethiopia (Approval/Permit Number: 002003, date of approval: 12 November 2024).

## Consent to participate

Written informed consent was obtained from the North Wollo Zone Education Department for obtaining access to the principals and teachers of the sample schools before the survey questionnaires were distributed. The consent from each participant was endorsed in the introduction part of the questionnaire, and oral consent was obtained during the orientation session, so they read, agreed, and filled out the questionnaire.

## Data Availability

Figshare: Leading beyond the Job Description: Investigating the Association between Principals’ Leadership Styles and Teachers’ Organizational Citizenship Behaviors in North Wollo Secondary Schools. https://doi.org/10.6084/m9.figshare.32660844 ( Yihenew B., 2026). The research contains the following underlying data:
• Data file 1. Datasets. (Anonymized answers to questionnaire in SPSS20). Data are available under the terms of the Creative Commons Attribution 4.0 International license (CC-BY 4.0).

## References

[ref1] Abdulrab M : Beyond leadership: How transformational leaders foster organizational citizenship and engagement through psychological empowerment. *J. Chin. Hum. Resour. Manag.* 2025;16(1):119–145. 10.47297/wspchrmWSP2040-800507.20251601

[ref2] Adnan M Manu P Yousaf M : Teachers' organizational citizenship behavior as a predictor of organizational success in the educational context of Pakistan. *Journal of Regional Studies Review.* 2026;V(1):94–188. 10.62843/jrsr/2026.5a174

[ref3] Akullo L Kamanyire R : Head Teacher Leadership Styles and Teacher Performance in Primary Schools in Kaabong District, Uganda. *J. Educ. Pract.* 2023;7(1):40–67. 10.47941/jep.1182

[ref4] Arar K Abu-Nasra M : Leadership style, organizational citizenship behavior, and school climate in Arab education systems. *J. Educ. Adm.* 2020;58(3):295–311. 10.1108/JEA-06-2019-0102

[ref5] Atış D Avcı F : Organizational Citizenship Behaviors and Positive Psychological Capital among Teachers within the eTwinning Professional Ecosystem. *Journal of Research in Social Sciences and Language.* 2026;6(1):378–395. 10.71514/jssal/2026.258

[ref6] Assefa TM : Educational leadership practices and challenges in secondary schools of Amhara Region, Ethiopia. *Ethiopian Journal of Education and Sciences.* 2024;19(2):45–61.

[ref7] Avolio BJ Bass BM : *Multifactor Leadership Questionnaire: Manual and Sampler Set.* 3rded: Mind Garden Inc;2004. https://www.mindgarden.com/16-multifactor-leadership-questionnare

[ref8] Bass BM : Theory of transformational leadership redux. *Leadersh. Q.* 1995;6(4):463–478. 10.1016/1048-9843(95)90021-7

[ref9] Bass BM Riggio RE : *Transformational leadership.* Lawrence Erlbaum Associates; 2nd ed. 2006. 10.4324/9781410617095

[ref10] Bledsoe MT : *Exploring the lived experiences of new elementary school administrators legislatively mandated to close the educational opportunity gap for students with challenging behavior: A phenomenological study.* ProQuest Dissertations and Theses Global'2024. (Doctoral dissertation).

[ref11] Constantinou P : Investigating the impact of transformational school leadership: Teacher perceptions and the role of leadership training programs. *Educ. Sci.* 2025;15(11):1495. 10.3390/educsci15111495

[ref12] Creswell J : *Research design: Qualitative, Quantitative, and Mixed Methods Approaches.* SAGE Publications, Inc.; 4th ed 2014.

[ref13] Creswell JW Creswell JD : *Research Design: Qualitative, Quantitative, and Mixed Methods Approaches.* Sage; 5th ed 2018.

[ref14] Daniels VA : *An investigation of summer reading performance and principals' perspectives on retention and assessment of third graders.* ProQuest Dissertations and Theses Global;2023. (Doctoral dissertation).

[ref15] Demeke G : *The historical development of education policy in Ethiopia.* Addis Ababa University Press;2011.

[ref16] Desta A : *Exploring organizational citizenship behavior among secondary school teachers in Ethiopia.* Bahir Dar University;2018. (Unpublished master's thesis).

[ref17] Elias O : *The impact of administrative leadership on secondary school teacher performance in the Amhara Regional State.* Addis Ababa University;2020. (Doctoral dissertation).

[ref18] Fang Z Yu S-C : Cross-level influence of group-focused transformational leadership on organizational citizenship behavior among Chinese secondary school teachers. *Behav. Sci.* 2023;13(10):848. 10.3390/bs13100848 37887498 PMC10604576

[ref19] Gedifew MT : The effect of transformational leadership on teachers' Organizational Citizenship Behavior in Amhara Regional State public universities, Ethiopia. *Cogent Education.* 2025;12(1):2556894. 10.1080/2331186X.2025.2556894

[ref20] Gougas V Malinova L : School Leadership. Models and Tools: A Review. *Open Journal of Social Sciences.* 2021;9:120–139. 10.4236/jss.2021.91009

[ref21] Hermanto YB Srimulyani VA Pitoyo DJ : The mediating role of quality of work life and organizational commitment in the link between transformational leadership and organizational citizenship behavior. *Heliyon.* 2024;10(6):e27664. 10.1016/j.heliyon.2024.e27664 38509945 PMC10950668

[ref22] Herren K : Does the Market or Clan Control Configuration Best Motivate Teachers to Demonstrate Organizational Citizenship Behavior? *ELPS.* 2014. http://hdl.handle.net/1808/18647.

[ref23] Ingersoll RM Dougherty P Sirinides P : School leadership and teacher retention. *Am. J. Educ.* 2018;124(2):141–166.

[ref24] Isacco A Morse JQ : Male graduate students at a “women’s college”: Examining the roles of academic motivation, support, connection, and masculinity ideology. *Sex Roles: A Journal of Research.* 2015;72(7-8):321–334. 10.1007/s11199-015-0447-3

[ref25] Johnson RB Onwuegbuzie AJ : Mixed Methods Research: A Research Paradigm Whose Time has come. *Educational Researcher.* 2004;33(7):14–26. 10.3102/0013189X033007014

[ref26] Johnson S O'Neill DK : Principals' Leadership and Teachers' Organizational Citizenship Behaviors. *Journal of Educational Administration.* 2014;52(2):180–202.

[ref27] Karabatak S Alanoglu M Sebgur D : The Effect of Teachers’ Organizational Citizenship Behaviours and Stress Levels on School Effectiveness. *European Journal of Educational Studies.* 2018;5(4):67–82. Publisher Full Text.

[ref28] Kearney SP : Leading organizational change in a Catholic school community. *School Leadership & Management.* 2026;46(2):246–270. 10.1080/13632434.2026.2630636

[ref29] Leithwood K Sun J : The Nature and Effects of Transformational School Leadership. *Educational Administration Quarterly.* 2012;48:387–423. 10.1177/0013161x11436268

[ref30] Le TN : Transformational leadership and innovation in Vietnamese higher education: The serial mediating roles of engagement and citizenship behaviour. *SA J. Hum. Resour. Manag.* 2026;24:1–9. 10.4102/sajhrm.v24i0.3326

[ref31] Liu Y Bellibas MS Gumus S : The effect of transformational leadership on teachers' organizational citizenship behavior: A multicultural study. *Educational Management Administration & Leadership.* 2024;52(1):112–134.

[ref32] Ludwikowska K Zakkariya KA Aboobaker N : Academic leadership and job performance: the effects of organizational citizenship behavior and informal institutional leadership. *Asian Education and Development Studies.* 2024;14(2):115–131. 10.1108/aeds-04-2024-0074

[ref33] Markos S Bezabih M : Predictors of organizational citizenship behavior in private higher education institutions in Ethiopia. *Journal of Higher Education Leadership and Management.* 2022;14(1):78–93.

[ref34] Maxcy SJ : Pragmatic Threads in Mixed Methods Research in the Social Sciences: the Search for Multiple Modes of Inquiry and the End of the Philosophy of Formalism. In Tashakkori A Teddlie C (Eds), Handbook of Mixed Methods in Social and Behavioral Research Sage; (2003).

[ref35] McIntyre CJ : Preferred style of leader among UK primary school teachers: Comparing social identity, transformational, transactional, and laissez-faire approaches. *Int. J. Leadersh. Educ.* 2026;29(2):1–26. 10.1080/13603124.2025.2609274

[ref36] Ministry of Education: *Education sector development program VI (ESDP VI) (2020/21–2024/25).* Federal Democratic Republic of Ethiopia;2023.

[ref37] Mugizi W Mwangi JK Karanja K : Transformational leadership and organizational citizenship behavior of teachers in selected primary schools. *Journal of Educational Research and Practice.* 2019;9(1):220–235.

[ref38] Niazi MF : *Exploring Organizational Citizenship Behavior in Government and Non-government Organizations.* Islamabad, Pakistan: Unpublished M.Sc. Research Report, National Institute of Psychology, Quaid-i-Azam University;2005.

[ref39] Organ DW : *Organizational citizenship behavior: The good soldier syndrome.* Lexington Books;1988.

[ref40] Organ DW : *Organizational citizenship behavior: Recent trends and future directions.* Frontiers in Psychology;2018.

[ref41] Öz H : The predictive power of organizational identification on teachers' organizational citizenship behaviors. *Front. Psychol.* 2026;17. 10.3389/fpsyg.2026.1818062 PMC13152760 42111573

[ref42] Pallant J : *SPSS survival manual: A step-by-step guide to data analysis using IBM SPSS.* (7th ed.): Routledge;2020.

[ref43] Rallis SF Rossman GB Mixed Methods in Evaluation Contexts: A Pragmatic Framework. In Tashakkori A Teddlie C (Eds), Handbook of Mixed Methods in Social and Behavioral Research. Sage Publications;2003.

[ref44] Sharma V Yadav R : Leadership styles and teacher citizenship behavior: A meta-analysis of educational environments. *Educ. Rev.* 2024;76(2):201–224. 10.1080/00131911.2024.2312245

[ref45] Temesgen B Melaku A : Practicing organizational citizenship behavior in primary schools of Bahir Dar: A qualitative inquiry. *Ethiopian Journal of Behavioral Sciences.* 2022;5(2):112–129.

[ref46] Tesfaye S : *Policy implementation bottlenecks in Ethiopian school leadership development.* ProQuest Dissertations and Theses Global;2018. (Doctoral dissertation).

[ref47] Tigist M Zenebe A Belete B : Validation of the Multifactor Leadership Questionnaire (MLQ-5X) in Ethiopian educational settings. *Ethiopian Journal of Education and Sciences.* 2023;18(2):45–62.

[ref48] Yihenew B : Leading beyond the Job Description: Investigating the Association between Principals’ Leadership Styles and Teachers’ Organizational Citizenship Behaviors in North Wollo Secondary Schools.Dataset. *figshare.* 2026. 10.6084/m9.figshare.32660844

[ref49] Yohannes ME Wasonga TA : Leadership styles and teacher job satisfaction in Ethiopian schools. *Educational Management Administration & Leadership.* 2021;51(5):1200–1218. 10.1177/17411432211041625

