# Supplementary material for: Leading beyond the Job Description: Investigating the Association between Principals’ Leadership Styles and Teachers’ Organizational Citizenship Behaviors in North Wollo Secondary Schools
Source: F1000Res. 2026 Oct 8;15:1186. Originally published 2026 Jul 20. [Version 3] doi: 10.12688/f1000research.183874.3 (PMC13560027; doi:10.12688/f1000research.183874.3)
Supplement: Peer review history

# Reviewer report: Reviewer response for version 2

**DOI:** 10.5256/f1000research.207954.r512586

**Published:** 2026-08-26

**Authors:** Zhuotao Fang

**Affiliations:** 1 Minzu Normal University of Xingyi, Xingyi, China

The authors have addressed all of my concerns satisfactorily. I have no further comments.

Is the work clearly and accurately presented and does it cite the current literature?

Partly

If applicable, is the statistical analysis and its interpretation appropriate?

Partly

Are all the source data underlying the results available to ensure full reproducibility?

Partly

Is the study design appropriate and is the work technically sound?

Yes

Are the conclusions drawn adequately supported by the results?

Partly

Are sufficient details of methods and analysis provided to allow replication by others?

Partly

Reviewer Expertise:

Educational Leadership and Management

I confirm that I have read this submission and believe that I have an appropriate level of expertise to confirm that it is of an acceptable scientific standard.

# Reviewer report: Reviewer response for version 2

**DOI:** 10.5256/f1000research.207954.r512585

**Published:** 2026-08-22

**Authors:** Mollaw Abreha

**Affiliations:** 1 Woldia University, Woldia, Ethiopia

The authors have adequately and satisfactorily addressed all the comments raised during the review process. The revisions have significantly improved the quality of the manuscript. Therefore, I recommend the manuscript for indexing in its current form.

Is the work clearly and accurately presented and does it cite the current literature?

Yes

If applicable, is the statistical analysis and its interpretation appropriate?

Yes

Are all the source data underlying the results available to ensure full reproducibility?

Yes

Is the study design appropriate and is the work technically sound?

Yes

Are the conclusions drawn adequately supported by the results?

Yes

Are sufficient details of methods and analysis provided to allow replication by others?

Yes

Reviewer Expertise:

Education, curriculum, gender, quality, etc.

I confirm that I have read this submission and believe that I have an appropriate level of expertise to confirm that it is of an acceptable scientific standard.

# Reviewer report: Reviewer response for version 1

**DOI:** 10.5256/f1000research.202959.r503689

**Published:** 2026-07-31

**Authors:** Zhuotao Fang

**Affiliations:** 1 Minzu Normal University of Xingyi, Xingyi, China

1. The manuscript claims to employ an explanatory sequential mixed-methods design (QUAN→qual), but the qualitative component contributes very little beyond confirming the quantitative findings. At present, the qualitative component appears supplementary rather than constituting a genuine explanatory mixed-methods study. The authors should clarify how interview participants were selected, how interview data were analyzed, whether thematic analysis or another analytic framework was used, how qualitative findings informed interpretation of quantitative results.

 2. The manuscript does not report any assessment of common method bias, please consider Harman’s single-factor test or CFA marker approaches.

 3. For language, although generally understandable, numerous language issues remain. For examples: “sows” instead of “shows”, inconsistent spacing around statistical notation, inconsistent reporting of p-values, “Laissez-fair” versus “Laissez-faire”.

 4. The manuscript states that 334 questionnaires were distributed but that 391 questionnaires were returned.

 5. Tables 4 and 6 show negative correlations and regression coefficients for laissez-faire leadership. However, the accompanying text sometimes reports these coefficients without the negative sign.

 6. A complete reference audit is required. Several citations appear unrelated to the statements for which they are used. For example: Johnson and Onwuegbuzie (2004) is a general mixed-methods paper but is cited as evidence that the MLQ has been validated in educational settings. Leithwood and Sun (2012) does not appear to provide a method for identifying missing values, outliers, and data errors. 

 7. I found more numerical inconsistencies and raise concerns about score construction and reliability of this study.

Is the work clearly and accurately presented and does it cite the current literature?

Partly

If applicable, is the statistical analysis and its interpretation appropriate?

Partly

Are all the source data underlying the results available to ensure full reproducibility?

Partly

Is the study design appropriate and is the work technically sound?

Yes

Are the conclusions drawn adequately supported by the results?

Partly

Are sufficient details of methods and analysis provided to allow replication by others?

Partly

Reviewer Expertise:

Educational Leadership and Management

I confirm that I have read this submission and believe that I have an appropriate level of expertise to confirm that it is of an acceptable scientific standard, however I have significant reservations, as outlined above.

# Response

**Published:** 2026-08-02

**Authors:** yihenew belay

Dear Reviewer,

 We would like to sincerely thank you for your thoughtful comments, comprehensive review, and constructive feedback on our manuscript.

 Your suggestions have greatly enhanced the clarity, methodological rigor, and overall quality of our work. We have carefully considered each point you raised and incorporated all revisions into the updated version of the manuscript. Once again, we are deeply grateful for your time and expertise, which have played a vital role in improving the quality of this paper. I have attached the responses.

 In response to the reviewer's request for methodological clarifications, the criteria for selecting interview participants and the specific analytical frameworks used for qualitative data have been addressed in the last paragraphs of sections 2.2 and 2.6. Furthermore, explanations of how the qualitative findings informed the quantitative interpretations have been integrated into sections 3.2, 3.3, and Part 4 under Research Question 1. Regarding the recommendation to apply Harman’s single-factor test or Confirmatory Factor Analysis (CFA) marker approaches, the manuscript already considers the CFA approach.

 Revisions were made to address the reviewer's concerns regarding language, formatting, and data reporting. Typographical and language issues—such as changing "sows" to "shows," correcting the spelling of "Laissez-faire," and standardizing the spacing around statistical notations and the reporting of p-values—have been fixed throughout the manuscript. Additionally, a typographical error concerning the sample size was corrected to indicate that exactly 319 questionnaires were returned, resolving the previous contradiction. The text corresponding to Tables 4 and 6 has also been revised to accurately report the negative signs for the correlation and regression coefficients associated with laissez-faire leadership.

 Finally, a complete reference audit was conducted, and issues regarding score construction and reliability were addressed. The citation for Johnson and Onwuegbuzie (2004) was removed from the Data Collection Instruments section (part 2.3) and is now properly retained in the research design section (part 2.1). Additionally, Leithwood and Sun (2012) was replaced with Tabachnick and Fidell (2019) to appropriately support the methodologies for handling missing values, outliers, and errors. To resolve concerns regarding numerical inconsistencies, validity, and reliability, further details have been added to section 2.4 of the revised manuscript, with a particular focus on expanding the qualitative methodology.

# Reviewer report: Reviewer response for version 1

**DOI:** 10.5256/f1000research.202959.r503684

**Published:** 2026-07-27

**Authors:** Mollaw Abreha

**Affiliations:** 1 Woldia University, Woldia, Ethiopia

The use of validated instruments, proper mixed-methods design, and open-science data sharing via Figshare all demonstrate the manuscript's outstanding academic rigor, solid theoretical foundation, and admirable transparency. Within an understudied regional context, the work effectively answers its research questions and contributes significantly to the literature. However, minor editorial corrections must be made before final indexing of article. Specifically, a thorough copyediting pass must be made to fix typographical errors like "sows" instead of "shows" and "QUAN→qual" instead of "QUAN!qual”.

Is the work clearly and accurately presented and does it cite the current literature?

Yes

If applicable, is the statistical analysis and its interpretation appropriate?

Yes

Are all the source data underlying the results available to ensure full reproducibility?

Yes

Is the study design appropriate and is the work technically sound?

Yes

Are the conclusions drawn adequately supported by the results?

Yes

Are sufficient details of methods and analysis provided to allow replication by others?

Yes

Reviewer Expertise:

Education, curriculum, gender, quality, etc.

I confirm that I have read this submission and believe that I have an appropriate level of expertise to confirm that it is of an acceptable scientific standard.

# Response

**Published:** 2026-07-28

**Authors:** yihenew belay

I received the reviewer's constructive comments and revised/corrected the two grammatical errors.
